# Effects of chronic stress on smartphone addiction: A moderated mediation model

**DOI:** 10.3389/fpubh.2023.1048210

**Published:** 2023-01-20

**Authors:** Huake Qiu, Hongliang Lu, Jiawei Pei, Yajuan Zhang, Yongjie Ma, Chen Xing, Xinlu Wang, Xia Zhu

**Affiliations:** ^1^Department of Military Medical Psychology, Air Force Medical University, Xi'An, China; ^2^Outpatient Department, 969 Hospital of PLA, Hohhot, China

**Keywords:** chronic stress, smartphone addiction, intolerance of uncertainty, emotion differentiation, moderated mediation model

## Abstract

**Introduction:**

Based on the compensatory Internet use theory and diathesis-stress model, the present study explores the effects of chronic stress on smartphone addiction (SPA). As intolerance of uncertainty and emotion-related variables are important factors that affect addictive behavior, we explore the mediating role of intolerance of uncertainty and the moderating role of emotion differentiation.

**Methods:**

We conducted a questionnaire survey of 286 participants (13.64% female; *M*_age_ = 22.88; *SD* = 3.77; range = 17–39) on chronic stress, SPA, intolerance of uncertainty, and emotion differentiation. SPSS 28.0 was used to analyze the descriptive statistics and correlations and test the moderated mediation model.

**Results:**

We find that (1) intolerance of uncertainty, SPA, and chronic stress are positively correlated with each other. Positive emotion differentiation is positively correlated with intolerance of uncertainty and negative emotion differentiation. (2) Intolerance of uncertainty plays a mediating role in chronic stress and SPA. (3) Positive emotion differentiation significantly moderates the relationship between chronic stress and SPA. Under the condition of low positive emotion differentiation, chronic stress is more effective in predicting SPA.

**Discussion:**

These findings may contribute to intervention and prevention programs for SPA. Thus, the intervention and prevention of SPA can start from two directions-reduce the intolerance of uncertainty and enhance the ability to experience positive emotion differentiation.

## 1. Introduction

With the progress and development of science and technology, the penetration rate of mobile phones in the population has increased from 33.9% in 2015 to 103.5% in 2017 (1). As a widely used medium among people, smartphone has brought many conveniences to people's lives. It has strengthened the connection between people (2), enriched daily entertainment, and improved people's life satisfaction and subjective happiness to a certain extent (3).

Excessive use of smartphones leads to smartphone addiction (SPA), also called problematic smartphone use (4–6), which is a type of behavioral addiction. Behavioral addiction is when individuals cannot control their desire for certain behaviors, leading to physical or psychological harm to themselves or others (7–9). Goodman (10) proposed that addiction has two aspects—repeated and uncontrollable behaviors—and it is difficult to stop the behavior even if it has significant negative effects on the individual (10). Although SPA has not been mentioned in The Diagnostic and Statistical Manual of Mental Disorders, Fifth Edition (DSM-5) and International Classification of Diseases 11th Revision (ICD-11), one study conducted an exploratory factor analysis and proved the similarity between SPA and substance-related addictive disorders in DSM-5, including compulsive behavior, functional impairment, withdrawal, and tolerance (11). Furthermore, gaming disorder has been included in ICD-11. Mobile games have made smartphones an important device for playing games, and addiction to games is a crucial factor that leads to SPA (12). Although online content can be carried out through various devices, the use of smartphones promotes the occurrence of Internet use disorder (5). The effect size of SPA associated with problematic social media use is medium to large because social media use is mostly through smartphones (13). In summary, SPA is an extremely important concept in exploring digital behavioral addictions as it intersects with many addictive behaviors mentioned above.

SPA is considered one of the crucial causes of human health problems in the information-based society (14). For physical health, SPA might induce neck and hands uncomfortableness (15, 16), and sleep quality would be affected by SPA, leading to low self-regulation and bedtime procrastination (17). Regarding mental health, after studying a large number of university students, Demirci et al. (18) found that SPA is closely related to anxiety and depression (18). Therefore, it is necessary to research the influencing factors of SPA.

For working adults, working during non-working hours and overtime work have become the norm in most professions, leading to great work pressure (19). In daily life, interpersonal communication and family relations have also brought great psychological burden to young people, such as bank loans and interpersonal conflicts. Chronic stress refers to constant and long-term stress (20). Chronic stress and acute stress are corresponding. The key to distinguishing the two concepts lies in the duration of exposure to stressors. The first exposure to stressors may induce acute stress reaction, and the stressors may become chronic stressors with an increase in exposure time and frequency (21).

According to the compensatory Internet use theory, people overuse technologies, such as the Internet or smartphones, to mitigate the negative effects they feel in life and work (22). Some studies have found that a smartphone is like an “adult pacifier.” Using a smartphone is considered a useful way of relieving pressure (3). Moreover, with the increase in work and life pressure of young people, the entertainment function of smartphones has received much attention, which has gradually extended the time of using smartphones, leading to SPA. SPA has a negative impact on mental health, making individuals have lower subjective and psychological wellbeing (23). However, subjective wellbeing is negatively correlated with perceived stress (24), so individuals are more inclined to use a smartphone to relieve stress (25). Therefore, chronic stress has a positive impact on SPA, and SPA, in turn, increases the pressure on individuals, thereby affecting their physical and mental health. However, there are few studies on the psychological mechanism between chronic stress and SPA, and the increasing phenomenon of SPA makes it extremely urgent to study the intervention of SPA. Therefore, it is essential to study the mechanism of the influence of chronic stress on SPA, and we put forward the following hypothesis:

**Hypothesis 1 (H1)**. *Chronic stress affects an individual's SPA, and the higher the chronic stress, the higher the SPA*.

Intolerance of uncertainty is one of the structures of a generalized anxiety disorder (26), which is closely related to worry (27) and refers to an individual's state when faced with ambiguous situations or stimuli (28). The relationship between intolerance of uncertainty and stress is complex, and the two can influence each other. Racial stress perceived by blacks can influence their state of worry, whereas intolerance of uncertainty can completely mediate the relationship between perceived racial stress and worry (29). A study on COVID-19 found that different personality traits have different intolerance of uncertainty, which affects the intensity of perceived stress (30). In addition, stress disorder is related to post-traumatic stress disorder, and the intolerance of uncertainty can predict the occurrence of post-traumatic stress symptoms (31). In summary, the above studies have demonstrated that stress and intolerance of uncertainty are closely related.

Numerous studies have revealed that intolerance of uncertainty affects SPA (32). Longitudinal studies suggest that the impact of intolerance of uncertainty on SPA is not entirely direct. Unsociable smartphone use is positively correlated with intolerance of uncertainty. Moreover, unsociable smartphone use mediates the intolerance of uncertainty and problematic smartphone use (33). Working remotely on the Internet during the COVID-19 pandemic has become mainstream. Intolerance of uncertainty increases people's pain (34), depression, and risk perception (35), which then increase their use of the Internet to ease pressure. Therefore, this study suggests that intolerance of uncertainty plays a mediating role in chronic stress and SPA. Therefore, we propose the following hypothesis:

**Hypothesis 2 (H2)**. *The influence of chronic stress on SPA is not entirely direct, and intolerance of uncertainty plays a mediating role in the relationship between them*.

The diathesis-stress model proposes that psychological state and coping styles in the face of pressure are different for subjects with different qualities (36). This indicates that not all people will be negatively affected by stress, and individual differences play an important role in coping with stress. Moreover, SPA may be one of the negative effects of chronic stress. According to this, some important abilities may moderate the negative effects of stress and play a key role in the relationship between chronic stress and SPA. In recent years, researchers put forward the diathesis-stress model of emotion differentiation and proved it through an interview study (37). Emotion differentiation refers to individual differences in emotional experience, which includes positive and negative emotion differentiation (38). Individuals with high emotion differentiation can better refine their perceived emotions, whereas individuals with low emotion differentiation can only describe experienced emotions in a general way. Individuals' perceived emotional states are associated with SPA. Negative emotion is significantly related to SPA (39). In addition, emotion regulation plays an important role in college students' SPA (40). Dysfunctional emotion regulation may lead to excessive smartphone use, contributing to problematic smartphone use (41). This suggests that an individual's ability for emotion differentiation may play a moderating role in the relationship between chronic stress and SPA.

The intolerance of uncertainty is closely related to an individual's emotional condition and emotion regulation ability. In adolescents with autism spectrum disorder, intolerance of uncertainty is influenced by emotion regulation, mediating emotion regulation, and symptoms of anxiety and depression (42). Negative emotion differentiation can mediate the relationship between stress and depression, and the lower the negative emotion differentiation, the stronger the predictive effect of stress on depression (37). Additionally, intolerance of uncertainty is closely related to depression (43), both of which have negative effects on stress. Therefore, based on the diathesis-stress model, the effect of chronic stress is influenced by individual diathesis (36). Thus, the relationship between chronic stress and intolerance of uncertainty may be affected by emotion differentiation, so the following hypothesis is proposed:

**Hypothesis 3 (H3)**. *Emotion differentiation regulates the relationship between chronic stress and SPA and its mediating mechanism. Thus, chronic stress has different relationships with SPA under different emotion differentiation conditions, and chronic stress has different relationships with intolerance of uncertainty, thus affecting SPA*.

**Figure 1:**
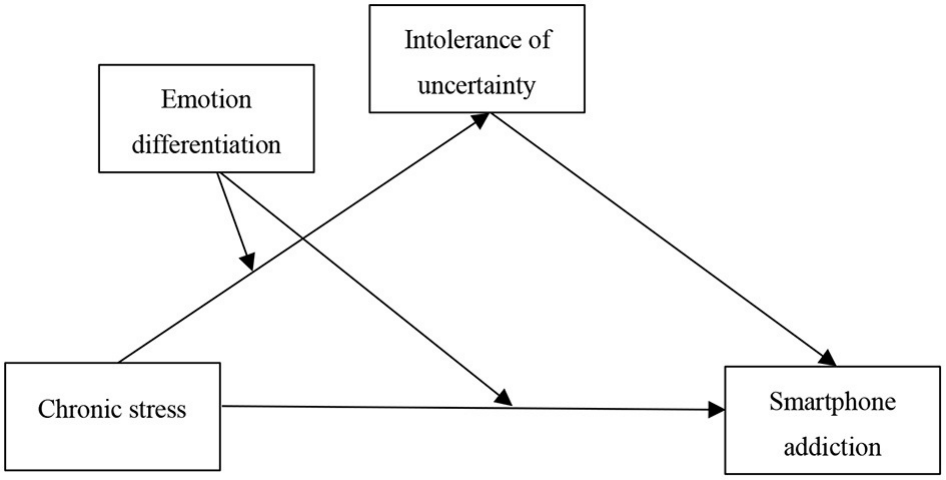
Hypothesized model.

Although many researchers have found a relationship between chronic stress and SPA, the psychological mechanism of how chronic stress affects SPA has not been investigated. From the perspective of the compensatory Internet use theory and the diathesis-stress model, the present study investigates whether chronic stress affects SPA and the mediating path and boundary conditions of chronic stress on SPA (see Figure 1).

## 2. Method

### 2.1. Participants

We randomly selected 293 enterprise employees from the northwest part of China. The questionnaires were answered by all participants. Participants who chose the same option in multiple scale questions in succession and spent too little time answering the questionnaires were excluded. A total of 286 participants (13.64% females; the participants' ages range from 17 to 39 years, with *M* ± *SD* =22.88 ± 3.77 years) who completed the questionnaires were used for the analysis. Among the participants, 72 (25.2%) had a high school degree or below; 104 (36.4%) had a junior college degree; 103 (36.0%) had a bachelor's degree; and 7 (2.4%) had a master's degree or above. The participants are right-handed, with normal intelligence and no dyslexia. They all volunteered to participate in the study and signed the informed consent.

### 2.2. Procedure

The questionnaires were distributed to all participants in the same period. An online network survey was adopted, and the questionnaires were administered through WeChat. To ensure the authenticity and accuracy of the research data, each participant could only answer the questionnaires once. After being informed of the purpose, cautions, and confidentiality of the study, a total of 293 participants completed a self-administered questionnaire. In the questionnaires, first, the participants provided their demographic information. Second, the participants filled out the Perceived Stress Scale (PSS), the Smartphone Addiction Scale (SAS), and the Intolerance of Uncertainty Scale. Finally, the ability of emotion differentiation was measured.

### 2.3. Materials

#### 2.3.1. Chronic stress

Chronic stress is assessed with PSS, which aims to measure participants' chronic stress intensity in the past month (44). It contains 14 items such as “In the last month, how often have you been upset because of something that happened unexpectedly?” Each item is rated on a five-point Likert scale, ranging from 0 (*never*) to *4* (*always*). Items 1, 2, 3, 8, 11, 12, and 14 are scored forward, where the higher the number, the greater the degree, whereas items 4, 5, 6, 7, 9, 10, and 13 are scored backward. The Cronbach's α of this scale is 0.75, and the construct validity is 0.88.

#### 2.3.2. SPA

SPA is assessed using the Short Version of SAS (SAS-SV) (9, 45). It contains 10 items such as “Missing planned work due to smartphone use.” Each item is rated on a six-point Likert scale ranging from 1 (strongly *agree*) to 6 (*strongly disagree*). All items are scored forward, with a higher number indicating a higher degree of SPA. The Cronbach's α for this scale is 0.92, and the construct validity is 0.90.

#### 2.3.3. Intolerance of uncertainty

A Chinese version of the Intolerance of Uncertainty Scale is used in this study, which has good reliability and validity when applied to the Chinese context (46–48). It contains 12 items such as “The unexpected makes me restless.” Each item is rated on a five-point Likert scale, ranging from 1 (*not at all*) to 5 (*extremely*). All items are scored forward, with a higher number indicating a higher degree of intolerance of uncertainty. The Cronbach's α for this scale is 0.90, and the construct validity is 0.90.

#### 2.3.4. Emotion differentiation

Following previous studies (49–51), we asked the participants to complete a standard laboratory-based emotion differentiation task. The participants viewed 20 negative and 20 positive images from the Open Affective Standardized Image Set (52) and rated a series of emotions on a 10-point scale, ranging from 1 (*not at all*) to 10 (*extremely*). Negative emotions (i.e., anger, ashamed, disgust, sadness, and scared) and positive emotions (i.e., calm, excitement, happiness, inspiration, and interested) were rated by the participants. Following prior work, each image was presented for 5 seconds, and the rating was self-paced.

The participants' negative emotion differentiation is investigated by calculating the average intraclass correlation coefficients (ICCs) of their ratings of 20 negative images. Lower ICCs indicate less similarity in how the participants use each emotion scale (51, 53). The final scores of ICCs are subtracted from one, so greater values represent higher emotion differentiation (54). The score of positive emotion differentiation is calculated in the same way.

### 2.4. Data analyses

All the data collected are processed using SPSS 28.0, which is used for descriptive statistics and correlation analysis. We take chronic stress as the independent variable, SPA as the dependent variable, intolerance of uncertainty as the mediating variable, and emotion differentiation as the moderating variable. PROCESS macro in SPSS 28.0 (55) is used to test the mediating and moderating effects. It is also used to explore the effect of chronic stress on SPA, the mediating role of intolerance of uncertainty, and the moderating role of emotion differentiation.

## 3. Results

### 3.1. Description and correlation

The descriptive statistics for each variable and the correlation analysis of the variables are presented in Table 1. The results of the correlation analysis indicate that SPA is positively associated with chronic stress. Intolerance of uncertainty, SPA, and chronic stress are positively correlated with each other. Moreover, the correlation coefficient between intolerance of uncertainty and SPA is moderate. In addition, positive emotion differentiation is positively correlated with intolerance of uncertainty and negative emotion differentiation.

**Table 1:** Descriptive statistics and correlations of all variables.

	***M***	***SD***	**1**	**2**	**3**	**4**	**5**
Chronic stress	21.65	7.52	1				
Smartphone addiction	18.14	9.29	0.34^***^	1			
Intolerance of uncertainty	27.75	9.64	0.33^***^	0.55^***^	1		
Negative emotion differentiation	0.70	0.29	−0.06	0.08	0.02	1	
Positive emotion differentiation	0.62	0.29	−0.06	0.06	0.15^*^	0.40^***^	1

M, mean; SD, standard deviations. ^*^p < 0.05; ^***^p < 0.001.

### 3.2. Examination of the mediation model

To reveal the influence mechanism of chronic stress on SPA, PROCESS macro (Model 4) in SPSS 28.0 is used to investigate the mediating role of intolerance of uncertainty in the relationship between chronic stress and SPA. The results of the mediating effect are presented in Tables 2, 3. The results in Table 2 indicate that chronic stress can significantly predict SPA (β = 0.33, *t* = 5.97, *p* < 0.001). After adding the mediating variables, it is found that both chronic stress (β = 0.17, *t* = 3.46, *p* < 0.001) and intolerance of uncertainty (β = 0.49, *t* = 9.50, *p* < 0.001) positively predict SPA.

**Table 2:** Mediation analysis.

**Regression equation**		**Overall fitting index**			**Regression coefficient**	
**Outcome variable**	**Predictive variable**	***R***	***R** ^2^*	***F(df)***	β	***t***
Intolerance of uncertainty		0.33	0.11	35.70${}_{\left(1\right)}^{***}$		
	Chronic stress				0.33	5.97^***^
Smartphone addiction		0.34	0.12	37.71${}_{\left(1\right)}^{***}$		
	Chronic stress				0.33	6.14^***^
Smartphone addiction		0.57	0.33	69.86${}_{\left(2\right)}^{***}$		
	Chronic stress				0.17	3.46^***^
	Intolerance of uncertainty				0.49	9.50^***^

All variables in the model were entered into the regression equation after standardization. ^***^p < 0.001.

**Table 3:** Testing the pathways of the mediation model.

	**β**	***SE***	**95% confidence interval**	
			**Lower**	**Upper**
Total effect	0.33	0.05	0.00	0.23
Direct effect	0.17	0.05	0.00	0.08
Indirect effect	0.16	0.03	0.10	0.23

To assess the significance of the indirect effect, bias-corrected bootstrap tests are performed using 5,000 samples at the 95% confidence interval, and the results are presented in Table 3. Intolerance of uncertainty has a significant indirect effect on the relationship between chronic stress and SPA (β = 0.16, *SE* = 0.03, *95% CI* = 0.10–0.23). The direct effect of chronic stress on SPA is also significant (β = 0.17, *SE* = 0.05, *95% CI* = 0.00–0.08).

### 3.3. Examination of the moderated mediation model

To reveal the mechanism of the effect of chronic stress on SPA, we use PROCESS macro (Model 8) in SPSS 28.0 to investigate the moderating effect of emotion differentiation in the relationship between chronic stress and intolerance of uncertainty, as well as between chronic stress and SPA. Negative emotion differentiation is used as a moderator variable, and the results reveal that the interaction of negative emotion differentiation and chronic stress has no significant predictive effect on intolerance of uncertainty (β = −0.01, *SE* = 0.06, *t* = −0.18, *p* = 0.85, *95% CI* = −0.13–0.11) and SPA (β = −0.03, *SE* = 0.05, *t* = −0.66, *p* = 0.51, *95% CI* = −0.14–0.07). When positive emotion differentiation is used as a moderator variable, the interaction between positive emotion differentiation and chronic stress has no significant predictive effect on intolerance of uncertainty (β = −0.03, *SE* = 0.06, *t* = −0.53, *p* = 0.60, *95% CI* = −0.14–0.08) and SPA (β = 0.03, *SE* = 0.06, *t* = 0.70, *p* = 0.48, *95% CI* = −0.06–0.13).

**Figure 2:**
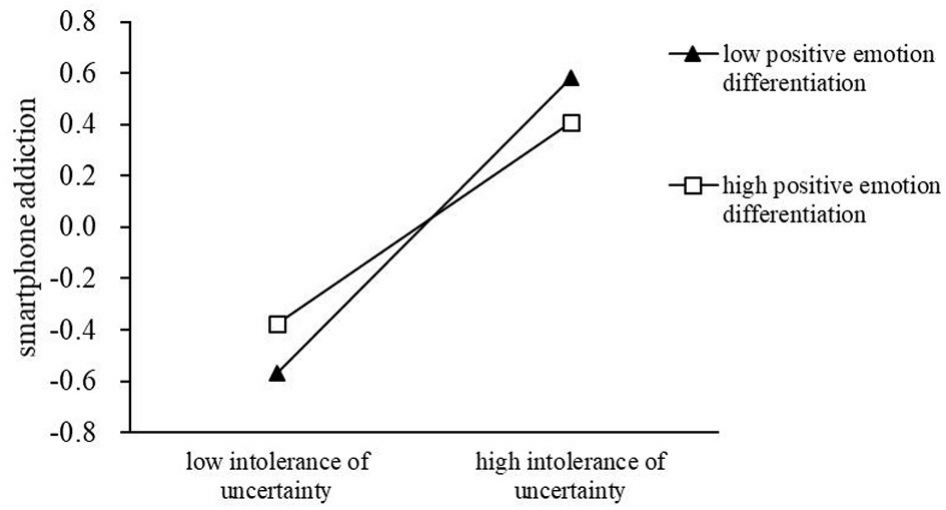
Simple slope plot of positive emotion differentiation.

The interaction between chronic stress and emotion differentiation is not significant in predicting intolerance of uncertainty and SPA. Therefore, PROCESS macro (Model 14) in SPSS28.0 is used to construct a moderating mediation model to examine whether emotion differentiation plays a moderating role in the relationship between intolerance of uncertainty and SPA. Negative emotion differentiation is used as a moderator, and the results reveal that the interaction of intolerance of uncertainty and negative emotion differentiation has no significant effect on SPA (β = −0.08, *SE* = 0.05, *t* = −1.72, *p* = 0.09, *95% CI* = −0.18–0.01). The moderating effect of positive emotion differentiation is presented in Tables 4, 5 and Figure 2. The results in Table 4 indicate that the interaction of intolerance of uncertainty and positive emotion differentiation has a significant negative predictive effect on SPA (β = −0.09, *SE* = 0.05, *t* = −2.01, *p* < 0.05, *95% CI* = −0.19–−0.00).

**Table 4:** Moderated mediation analysis.

**Regression equation**		**Overall fitting index**			**Regression coefficient**	
**Outcome variable**	**Predictive variable**	***R***	***R** ^2^*	***F(df)***	β	***t***
Intolerance of uncertainty		0.33	0.11	35.70${}_{\left(1\right)}^{***}$		
	Chronic stress				0.33	5.97^***^
Smartphone addiction		0.58	0.34	36.19${}_{\left(4\right)}^{***}$		
	Chronic stress				0.17	3.34^***^
	Intolerance of uncertainty				0.49	9.43^***^
	Positive emotion differentiation				0.00	0.07
	Positive emotion differentiation*intolerance of uncertainty				−0.09	−2.01*

All variables in the model were entered into the regression equation after standardization. ^*^p < 0.05; ^***^p < 0.001.

**Table 5:** Moderating effect of different positive emotion differentiation.

	**β**	***SE***	**95% confidence interval**	
			**Lower**	**Upper**
High positive emotion differentiation	0.13	0.03	0.07	0.20
Low positive emotion differentiation	0.19	0.04	0.11	0.28

According to Table 5, both low positive emotion differentiation and high positive emotion differentiation have different predictive effects on SPA. Low positive emotion differentiation (β = 0.19, *SE* = 0.04, *95% CI* = 0.11–0.28) is more predictive of SPA than high positive emotion differentiation (β = 0.13, *SE* = 0.03, *95% CI* = 0.07–0.20).

**Figure 3:**
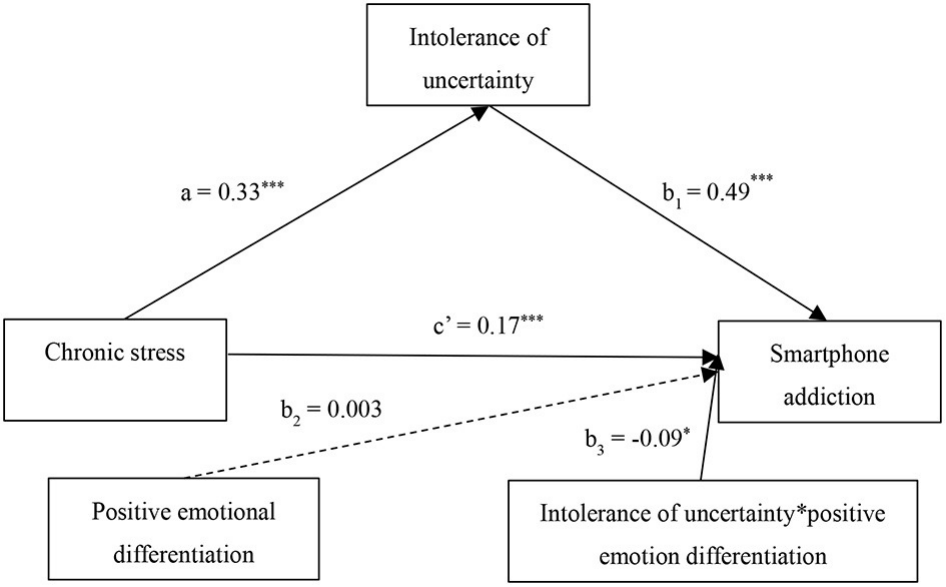
Statistic model.

Figure 2 depicts the results of a simple slope analysis. Compared with a high emotion differentiation condition (β = 0.40, *SE* = 0.07, *t* = 5.77, *p* < 0.001, *95% CI* = 0.26–0.53), in a low emotion differentiation condition, intolerance of uncertainty has a greater positive predictive effect on SPA (β = 0.58, *SE* = 0.07, *t* = 8.29, *p* < 0.001, *95% CI* = 0.45–0.72). Figure 3 depicts the statistical model of this study.

## 4. Discussion

### 4.1. The relationship between the dimensions

Through correlation analysis, this study initially finds that chronic stress, SPA, and intolerance of uncertainty are positively correlated with each other. Consistent with previous findings, stress is a key factor in the emergence, development, and relapse of addictive behaviors (56, 57). Stress promotes excessive eating behavior, and adapting to stress and reward circuit promotes metabolic adaptation, which affects eating addiction behavior (56). With the development and popularization of the Internet, studies have found that gaming disorder is closely related to stress (58). In addition, stress is closely related to intolerance of uncertainty. Intolerance of uncertainty predicts the extent of post-traumatic stress symptoms associated with negative stressful life events (59). There is also a strong relationship between intolerance of uncertainty and addictive behavior, and patients treated with opioids have higher intolerance of uncertainty (60). Therefore, the preliminary findings of this study indicate that there may be a complex relationship among the three variables, and we construct the relationship model among them.

### 4.2. The mediating role of intolerance of uncertainty

This study finds that chronic stress affects SPA through intolerance of uncertainty. The results of this study are consistent with those of other studies. Studies have demonstrated that chronic stress has a negative impact on mental health (61). Intolerance of uncertainty is closely related to worry, which closely reflects negative psychological wellbeing (27). Based on the ego depletion theory, stress promotes an individual's self-control to maintain the balance between the external pressure environment and their psychological wellbeing (62, 63). However, excessive self-control leads to self-depletion, psychological imbalance, or decline in self-control, which may have a negative impact on individuals (64).

The compensatory Internet use theory reveals that people release negative emotions and psychological pressure through the use of smartphones or the Internet (22). Intolerance of uncertainty is an important negative psychological feeling, and individuals can use smartphones or the Internet to alleviate negative psychological feelings. Therefore, the results of this study confirm the positive predictive effect of chronic stress on SPA and the mediating effect of intolerance of uncertainty on chronic stress and SPA.

### 4.3. The moderating role of emotion differentiation

The results of this study reveal that positive emotion differentiation plays a moderating role in the relationship between intolerance of uncertainty and SPA. Intolerance of uncertainty under low-level emotion differentiation is a greater positive predictor of SPA. The result of this study is consistent with that of other studies. Addicts have lower emotional wellbeing and intelligence, including emotion differentiation, than non-addicts (65, 66). Compared with non-alcoholics and abstainers, alcoholics have more difficulty in recognizing and expressing their feelings and have a lower emotion differentiation (67). Moreover, intolerance of uncertainty is a negative psychological state of individuals, which is closely related to their emotions. Therefore, individuals with low emotion differentiation are more vulnerable to the impact of intolerance of uncertainty, leading to SPA.

However, this study does not find the moderating effect of negative emotion differentiation. This could be because the original purpose of smartphone use is to seek positive emotions, such as happiness (3). Therefore, better recognition and expression of positive feelings can help individuals find the negative impact of positive emotions in the use of smartphones. This can help them avoid SPA caused by the excessive use of smartphones.

This study does not confirm H3, finding that neither positive nor negative emotion differentiation moderates chronic stress as a predictor of intolerance of uncertainty or SPA. Most studies have found the moderating role of negative emotion differentiation in chronic stress. High emotion differentiation alleviates anxiety and depression after exposure to stressful life events in adolescence (50). Rumination and constant attention to daily life are more strongly associated with depressive symptoms in individuals with low emotion differentiation (68). These findings are not consistent with the conclusion of this study. This may be because chronic stress reflects the degree of an individual's perceived stress in a certain period, whereas intolerance of uncertainty is an individual's negative psychological feeling, reflecting the negative emotions individuals feel when they are stressed. Moreover, emotion differentiation is the ability to recognize and distinguish emotions and can better adjust the influence of variables reflecting emotions. Further, the existing literature lacks mediating mechanism studies on the influence of chronic stress, but this study explores the mediating effect of chronic stress on SPA and further investigates the moderating effect of emotion differentiation. Therefore, the results of this study expand the research on the effects of chronic stress, and it is found that the moderating effect of emotion differentiation on the effects of chronic stress is mainly reflected in the negative effects.

### 4.4. Limitations and future research

There are several limitations in the present study. First, in the working environment, the relationship between leaders and employees, as well as leadership style, may have an important impact on the psychological feelings and behavior of employees (69, 70). Therefore, future studies can include relevant variables to explore their important role in individual psychological feelings and behavior to construct structural equation models. Second, the research method of this study is mainly a subjective assessment. This makes the results of the study subjective and subject to response bias. Thus, implicit behavioral research methods or cognitive neuroscience methods should be considered in future research to improve the objectivity and credibility of the study.

### 4.5. Strengths and implications

Despite these limitations, this study has the following strengths. First, it investigates the indirect effect of chronic stress on SPA, whereas previous studies mainly investigated the direct effect of chronic stress on SPA. Second, the important role of emotion differentiation in SPA is proposed for the first time. Third, the mechanism of positive emotion differentiation is disclosed by exploring the influences of both negative and positive emotion differentiation on SPA.

This study has significant implications. First, it is the first to examine the mediating role of intolerance of uncertainty in the relationship between chronic stress and SPA. It is revealed that the effect of chronic stress on SPA is indirect through intolerance of uncertainty. Second, the study finds that emotion differentiation plays a moderating role in the effect of chronic stress on SPA, providing support for future prevention and intervention. Individuals with high levels of positive emotion differentiation are less likely to suffer from chronic stress, thereby reducing the degree of SPA. Therefore, in future practice, cognitive behavior therapy or emotion regulation strategies can be used to reduce the intolerance of uncertainty that individuals feel when facing various types of pressure (42, 71). In addition, based on the moderating effect of emotion differentiation, mindfulness and other methods can be used to improve positive emotion differentiation (72), learn to better identify and express emotions, and reduce SPA.

## 5. Conclusion

In conclusion, the present study proposes a moderated mediation model to explain the effect of chronic stress on SPA and its mechanism. Specifically, chronic stress can significantly predict SPA, and intolerance of uncertainty plays a mediating role in the relationship between them. High chronic stress leads to high intolerance of uncertainty, resulting in SPA. In addition, positive emotion differentiation moderates the relationship between intolerance of uncertainty and SPA. Individuals with low positive emotion differentiation are more vulnerable to intolerance of uncertainty, leading to SPA.

## Data availability statement

The original contributions presented in the study are included in the article/supplementary material, further inquiries can be directed to the corresponding author.

## Ethics statement

Written informed consent was obtained from the individual(s) for the publication of any potentially identifiable images or data included in this article.

## Author contributions

HQ: conceptualization, methodology, writing–original draft, and writing–review and editing. HL: conceptualization, validation, writing–review and editing, and investigation. JP, YZ, YM, and XW: investigation. CX: software. XZ: supervision, writing–review and editing, and investigation. All authors contributed to the article and approved the submitted version.
